# Minimum acceptable diet practice and its associated factors among children aged 6–23 months in rural communities of Goncha district, north West Ethiopia

**DOI:** 10.1186/s40795-021-00444-0

**Published:** 2021-07-20

**Authors:** Bamlaku Birie, Andargachew Kassa, Emnet Kebede, Bezabih Terefe

**Affiliations:** 1 Department of Midwifery, College of Medicine and Health science, Mizan Tepi University, Mizan Aman, Ethiopia; 2 Department of Midwifery, College of Medicine and Health Science, Hawassa University, Awassa, Ethiopia; 3 Department of Midwifery, College of Medicine and Health Science, Arba Minch University, Arba Minch, Ethiopia

## Abstract

**Background:**

After the first 6 months breast milk is no longer sufficient to meet the nutritional needs of the infant. Therefore, complementary foods should be added to the child’s diet. Feeding children with a diversified diet is practiced improperly in developing countries including Ethiopia particularly in the rural community of the Amhara region. However, limited information was documented on the rural communities and no data were available specifically in the study area to show the exact picture of child feeding practices. So, this study was planned to assess minimum acceptable diet practice and its associated factors among children aged 6–23 months in the rural community of Goncha district, Amhara region, Ethiopia.

**Methods:**

Community-based cross-sectional study was employed to determine minimum acceptable diet practice and its associated factors among children aged 6–23 months at rural communities of Goncha district, East Gojjam zone, Amhara region, Ethiopia. A multi-stage sampling technique was used to select study subjects, and an interview administered structured questionnaire was used to collect the data. Data were entered by Epi Data version 4.0.2 and exported to SPSS 20 for analysis. Bivariate and multivariable logistic regression analysis was used to see the association. Then, *P*-value < 0.05 with 95% CI on multivariable logistic regression analysis were used to identify the predictor of the outcome variable.

**Results:**

A total of 430 mothers who have children aged 6–23 months were included in the analysis with a 98% of response rate. About 12.6% of children aged 6–23 months received the recommended minimum acceptable diet. Children whose mothers who had formal education [AOR = 2.7, 95%CI (1.133, 6.231)], institutional delivery [AOR = 4.5, 95%CI (1.986, 10.362)], media exposure [AOR = 2.6, 95%CI (1.303, 5.291)] and higher household wealth index [AOR = 2.5, 95%CI (1.139, 5.90)] were significantly associated with minimum acceptable diet.

**Conclusion:**

The practice of minimum acceptable diet in the study area was inadequate and very low according to the national recommendation. So, strengthening institutional delivery, improving the wealth of the community and exposure to media, and finally empowering women’s’ for education is recommended.

## Introduction

Sufficient nutrition in the earlier months of life is key to health and growth, and its importance goes throughout life [1]. Later six months breast milk is no longer adequate to meet the nutritional needs of the infant. Therefore, additional foods should be introduced to the child’s diet, which is the transition from exclusive breastfeeding to family foods. This is the most critical period because children are most susceptible to malnutrition during this transition [2].

A minimum acceptable diet (MAD) is an indicator for evaluating child feeding practices presented via World Health Organization. It is a combination of the minimum dietary diversity and minimum meal frequency [3]. Infants and young children should have a minimum acceptable diet (MAD) to ensure appropriate growth and development, otherwise, they are vulnerable to undernutrition especially stunting and micronutrient deficiencies, and increased morbidity and mortality [1, 4]. In addition to a lack of adequate and balanced diet, several people are attacked by diseases that occur due to protein-energy malnutrition and due to lack of disease-protecting foods. Children, pregnant women, and lactating mothers are most vulnerable to this problem [5].

Also, it is known that nutrition is one of the vital pre-conditions for achieving sustainable development goals because, from 17 SDGs, the first three icons are directly related to nutrition [6].

Although Ethiopia is a manufacturer of a diversity of agricultural products, still the countries are known in the world with the highest number of malnourished populations. Malnutrition is primarily seen among rural residents and the prevalence among children was 48.5% [7]. The Ethiopian government has been tried to improve child feeding practices by implementing the national nutrition program of child feeding practices and a multi-sectoral plan of nutrition intervention to end child undernutrition in Ethiopia by 2030 [8]. Despite this, the progress was not satisfactory, particularly the national prevalence of MAD practice was 7%. The burden is higher in rural areas and significant variation also exists between regions. Children in urban areas (19%) are more likely to feed than those in rural areas (6%) and the proportion of children who receive the minimum acceptable diet is highest (27%) in Addis Ababa and lowest (2%) in Amara region [2, 9].

Even though studies were conducted about the determinants of the optimal complementary feeding practices in Ethiopia, however, inadequate efficient information was documented about minimum acceptable diet practice and its associated factors independently and most of the studies were not representative especially for rural communities. Also, as far as the researcher’s knowledge is concerned, no documented data were accessible specifically in the study area. Therefore, this study was planned to assess minimum acceptable diet practice and its associated factors among children aged between 6 and 23 months in rural communities of Goncha district, Amhara region, Ethiopia during the year 2020. Finally, the findings of this study will help to identify high-risk groups in the formulation of appropriate complementary feeding interventions.

## Methods

### Study area and period

A community-based cross-sectional study was employed from June 15 to July 152,020 in rural communities of Goncha district, which is located in East Gojjam Zone, Amhara region, Ethiopia. The woreda (administrative division in Ethiopia) is located 154 km East of Bihar Dar, the capital city of the Amhara region, and 335 km far from the Northwest of Addis Ababa. This woreda is administratively structured by 43 Keble’s (lowest administrative division in Ethiopia) 41 rural and 2 urban Keble’s. Of these rural Keble’s 12 are lowlands and 29 are highlands. Almost all of the district’s population consists of subsistence farmers heavily depending on rains for their agriculture. The district is one of the areas in the region, known to experience chronic food insecurity due to variable rainfall pattern, and the population especially who are resided in lowland Keble’s are users of government safety net program. In the woreda, there are 8 governmental health centers with the ratio of one health center to five Keble’s and 43 health posts. Goncha woreda is settled by total populations of 210,423, of these 108,909 are females and 42,569 are in the reproductive age group (15–49). Out of the total population 28, 491 are under-five children’s and 10,626 (5% of the total population) are infants and young children aged 6–23 months (Goncha woreda health office annual report, 2012 E.C).

### Study participants

Children aged 6–23 months with mothers who are residents of the selected Keble’s at least 6 months and available during the data collection period were included. Children of mothers who were seriously ill during the data collection period and children having acute illness and other conditions which disturb appetite during the survey (determined by respondents’ self-report and by observation) were excluded.

### Sample size determination

The sample required for this study was calculated using single population proportion formula; by considering the following assumptions: Z = standard normal distribution (Z = 1.96) with a confidence interval of 95%, *p* = 8.6% taken from a proportion of MAD in Dembecha district [10], d = tolerable margin of error (d) =0.038, by adding 2 design effect and 5% non-response rate, the total sample size required for this study was = 439. To ensure the adequacy of sample size, Epi info was used to calculate sample size for factors associated with minimum acceptable diet.

### Sampling procedure

**Fig. 1:**
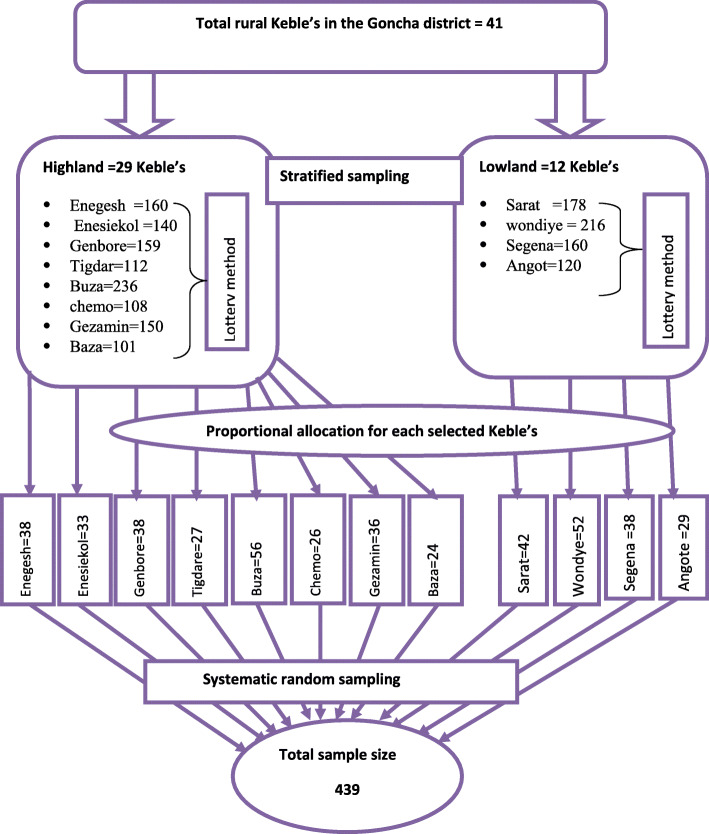
Schematic presentation of sampling procedure from total rural Keble’s, in Goncha district, Northwest Ethiopia, 2020

A multi-stage sampling technique was used to select the study subjects. First total rural Keble’s in Goncha district were stratified into highland (dega) and lowlands (kola) based on their predominating agro-ecological characteristics (classification obtained from Goncha woreda health office). Second, from the two strata, 8 from 29 highland Keble’s and 4 from 12 lowlands Keble’s were selected by using the lottery method. Then, the total sample size was allocated proportionally and sampling interval- “K” was determined. Finally, 439 children aged 6–23 months were selected by using a systematic sampling method based on the sampling frame obtained from Health Extension Worker’s (HEW’s) record. After randomly identified the first child, we preceded the second child every 4 intervals (Fig. 1).

### Dependent variable

Minimum acceptable diet.

### Operational definitions

#### Minimum acceptable diet

Proportion of children’s aged 6–23 month who had at least minimum meal frequency and minimum diversified diet during the previous day [11].

#### Minimum meal frequency

Proportion of breastfeeding and non-breastfeeding children aged 6–23 months who receive soft, solid, and semi-solid foods (but also including milk feeds for non-breast feed children) in the last 24 h. Breastfeed infants aged 6–8 months 2 times in the last 24 h; breastfeed infants and young children aged 9–23 months 3 times in the last 24 h. For non-breastfeeding infants and young children aged 6–23 months at least 4 times in the last 24 h [3].

#### Minimum dietary diversity

Proportion of children aged 6–23 months who receive five or more food groups out of the eight food groups in the last 24 h. These foods groups used for this indicator are breast milk, grains, roots and tubers; legumes and nuts; dairy products (milk, yogurt); Flesh foods (meat, fish, poultry, and liver/organ meats); eggs; vitamin A-rich fruits and vegetables; and other fruits and vegetables. Quality and quantity of any amount from those groups can be considered as sufficient to count [11].

#### Maternal knowledge on IYCF practice

Knowledge of mothers on infant and child feeding practice was measured based on ten knowledge questions. Each correct answer (yes) earned one point, and any wrong answer (no) got zero. The calculated knowledge score ranged from 0 to 10 points. Those who score above the mean (5.7 ± 2.6 standard deviations) was categorized as knowledgeable and those who score below the mean was categorized as not knowledgeable [12].

#### Exposure to media

Media exposure was categorized as satisfactory or unsatisfactory. Mothers who listen to the radio, or watched television at least once a week was satisfactory media exposure, and otherwise unsatisfactory [13].

### Data collection and quality control

Primary data was collected using interviewer-administered and structured questionnaires from selected children of mothers through a face-to-face interview. 8 data collectors and 2 supervisors have participated in the survey. Questionnaires were prepared in English and were translated into the local language (Amharic), and again it was translated back into English to keep its consistency. Data collectors were trained for one day before the data collection period. The collected data was checked regularly by supervisors and principal investigators for its completeness and consistency.

### Data processing and analysis

Data were entered by using Epi Data entry client version 4.0.2 and exported to SPSS 20 statistical package for analysis. Data cleaning was performed to check for consistencies and values. Dietary diversity score was computed out of eight from eight food groups, and household economic status was measured by constructing wealth index through principal component analysis (PCA) and ranked as low, middle, and high wealth index. Tables and graphs were used for data presentation. Bivariate logistic regression analysis was done to see the association of independent variables on the dichotomous outcome variable. To control the effect of potential confounders and to identify the independent effect of the explanatory variable on the minimum acceptable diet, variables with a *p*-value less than 0.25 in bivariate logistic regression were considered as a candidate for multivariable logistic regression analysis. Before going on multivariable logistic regression analysis multicollinearity was checked by co-linearity diagnostic tests and variables with variance inflation factor (VIF} less than ten, and variables that fulfill sample size assumptions were entered into the model. At this level, model fitness was checked with Hosmer and Lemeshow goodness of fit at a *p*-value ≥ of 0.05. Finally, variables with a p-value less than 0.05 and 95% CI were considered as the predictive for the outcome variable. The strength of associations and statistical significances between independent variables and outcome variables were expressed using OR and 95% confidence interval respectively.

## Result

### Socio-demographic characteristics

Among a total of 439 sampled subjects, 430 children aged between 6 and 23 months with mothers were enrolled in the study making a response rate of 98%. Among children aged 6–23 months, 217 (50.5%) were females and 167(38.8%) were categorized in the age group between 18 and 23 months. The mean age of children was 15.3 ± 5.4 (SD) months, and half of the children aged 6–23 months were in the birth order of second to fourth 218(50.7%). Concerning the educational status of children’s parents, About 283 (65.8%) and 262(60.9%) of mothers and fathers had no formal education respectively. From total children parents, about three fourth 320 (74.4%) of fathers were farmers followed by merchants 56(13.0%). (Table 1).

**Table 1:** Parental level socio-demographic characteristics of children aged 6–23 months, Goncha, Northwest Ethiopia, 2020 (*n* = 430)

Characteristics	Category	Frequency(n)	Percentage (%)
Mother age (years)	15–24	121	28.1
	25–34	206	47.9
	35–49	103	24.0
Marital status	Married	365	84.9
	Divorced	28	6.5
	Single	25	5.8
	Other^a^	12	2.8
Mother education	No formal education	283	65.8
	Formal education	147	34.2
Father education	No formal education	262	60.9
	Formal education	168	39.1
Mother Occupation	Farmer	173	40.2
	Housewife	182	42.3
	Merchant	56	13.0
	Other^b^	19	4.5
Father Occupation	Farmer	320	74.4
	Merchant	56	13.0
	Employee	40	9.3
	Other^c^	14	3.3
Sex of a child	Male	213	49.5
	Female	217	50.5
Age of child in month	6–11	123	28.6
	12–17	142	33.0
	18–23	165	38.4
Birth order of a child	First	132	30.7
	Second to fourth	218	50.7
	Above fourth	80	18.6
Agroecology	Dega	230	53.5
	Kola	200	46.5

^**a**^**died/separated,** ^**b**^**labor work/no current work/employer,** ^**c**^**labor work/no current work**

### Maternal and child health care utilization

During the pregnancy of sampled child, less than half 181(42.1%) of mother attended four and above times antenatal care visits, and more than half 262(60.9%) of mothers did not utilize postnatal care service. Out of the total children aged 6–23 months, the majority (60.5%) of children were delivered at home, and about a quarter 113(26.3%) of children aged 6–23 months did not start vaccination. Nearly all, 426(99.1%) of children aged 6–23 months were breastfed at the time of data collection, and around one-third, 148(34.4%) of 6–23 month aged children were started complementary food after six months. Greater than three fourth, 341(79.3%) of children did not receive monthly growth monitoring and promotion service. (Table 2).

**Table 2:** Maternal and child healthcare-related characteristics of children aged 6–23 month, Goncha, Northwest Ethiopia, 2020 (n = 430)

Characteristics	Frequency(n)	Percentage (%)
Antenatal service		
Fourth and above visit	181	42.1
One to three visit	117	27.2
No visit	132	30.7
Place of delivery		
Home	260	60.5
Health facility	170	39.5
Post natal care		
Within 7 day	44	10.2
After 7 days	124	28.9
No visit	262	60.9
Pre lacteal feeding		
Yes	207	48.1
No	223	51.9
Vaccination status		
Vaccinated/started	317	73.7
Not started	113	26.3
first, breastfeed for a child		
Within 1 h	156	36.3
After 1 h	274	63.7
Current Brest feeding status		
Breastfeeding	426	99.1
Not breastfeeding	4	.9
Time of starting complementary food		
Before 6 months after delivery	87	20.2
At 6 months	147	34.2
After 6 months	148	34.4
I do not know	48	11.2
Growth monitoring and promotion service		
No	341	79.3
Yes	89	20.7

### Mothers knowledge on child feeding practice and household level related characteristics

Concerning the knowledge of mothers on infant and young child feeding practice, greater than half, 253(58.8%) of mothers were knowledgeable. Out of the total study participants, one-third 138(32.1%) of children were living in houses where family members greater than five. Of the mothers of children aged 6–23 months, two-thirds 284(66%) of mothers participated in the household decision, and about one-third 140(32.6%) of mothers had satisfactory media exposure (see Table 3).

**Table 3:** maternal/caregiver knowledge on child feeding and household level related characteristics of children aged 6–23 months, Goncha, Northwest Ethiopia, 2020 (*n* = 430)

Characteristics	Frequency(n)	Percentage (%)
Maternal knowledge of IYCF		
Knowledgeable	253	58.8
Not knowledgeable	177	41.2
Family size		
> 5	138	32.1
< =5	292	67.9
No of under-five children within a household		
Two and above	150	34.9
One	280	65.1
Media exposure		
Unsatisfactory	290	67.4
Satisfactory	140	32.6
Mother participation in household decision		
Mother not involved	146	34.0
Mother involved	284	66.0
Wealth index		
Low	146	34
Middle	142	33
High	142	33

### Minimum acceptable diet practice

From total children aged 6–23 months, about 54(12.6%) of children meet the recommended minimum acceptable diet.

### Factors influencing minimum acceptable diet practice

To identify factors associated with minimum acceptable diet practice, bivariate and multivariable logistic regression analyses were done. On binary logistic regression analysis variables with *p*-value, less than 0.25 were considered as a candidate for multivariable logistic regression analysis. Finally, variables with a p-value less than 0.05 and 95% CI i.e. mother education, place of delivery, exposure to media, and wealth index were found to be the potential predictors of meeting minimum acceptable diet (See Table 4).

**Table 4:** Bivariate and multivariable logistic regression output showing factors associated with minimum acceptable diet practice among children’s aged 6–23 months, Goncha, Northwest Ethiopia, 2020, (n = 430)

Variable	Category	Not meet MAD	Meet MAD	COR (95%CI)	AOR (95%CI)
		N (%)	N (%)		
Agro ecology	Dega	196 (85.2)	34 (14.8)	1.56 (0.867–2.811)	0.8 (0.374–1.687)
	Kola	180 (90)	20 (10)	1	1
Place of delivery	Home	249 (95.8)	11 (4.2)	1	1
	Health facility	127 (74.7)	43 (25.3)	7.6 (3.82–15.37)**	4.5 (1.98–10.36)**
Pre lacteal fed for a child	No	188 (84.3)	35 (15.7)	1	1
	Yes	188 (90.8)	19 (9.2)	0.54 (0.30–0.983)*	1.28 (0.618–2.664)
PNC service	No	236 (90.1)	26 (9.9)	1	1
	Yes	140 (83.3)	28 (16.7)	1.8 (1.023–3.221)*	0.7 (0.329–1.488)
Mother education	No formal education	268 (94.7)	15 (5.3)	1	1
	Formal education	108 (73.5)	39 (26.5)	6.4 (3.41–12.18)**	2.7 (1.133–6.231)*
Father education	No formal education	245 (93.5)	17 (6.5)	1	1
	Formal education	131 (78)	37 (22)	4.07 (2.20–7.50)**	1.6 (0.71–3.677)
Family size	> 5	126 (91.3)	12 (8.7)	1	1
	<=5	250 (85.6)	42 (14.4)	1.76 (0.897–3.469)	1.4 (0.613–3.098)
Decision making in household	Mother not involved	132 (90.4)	14 (9.6)	1	1
	Mother involved	244 (85.9)	40 (14.1)	1.54 (0.811–2.944)	1.2 (0.564–2.528)
Exposure to media	Unsatisfactory	270 (93.1)	20 (6.9)	1	1
	Satisfactory	106 (75.7)	34 (24.3)	4.3 (2.385–7.86)**	2.6 (1.303–5.29)**
Knowledge of mothers on IYCF practice	Not knowledgeable	169 (95.5)	8 (4.5)	1	1
	Knowledgeable	207 (81.8)	46 (18.2)	4.7 (2.157–10.2)**	1.6 (0.626–3.954)
Wealth index	Low	132 (90.4)	14 (9.6)	1	1
	Middle	130 (91.5)	12 (8.5)	0.71 (0.32–1.61)	1.1 (0.445–2.72)
	High	114 (80.3)	28 (19.7)	2.7 (1.193–6.766)*	2.5 (1.139–5.90)*

Notice * p-value < 0.05, **p-value < 0.01, COR- crude odds ratio, AOR- adjusted odds ratio,

## Discussion

The finding of this study revealed that 12.6% with 95% CI (9.5, 15.7) of children aged 6–23 months were received the recommended minimum acceptable diet. This was higher compared to a study conducted in Northwest Ethiopia, Dembecha (8.6%) [10]. The variation might be due to the different study periods. The above study was conducted in populations where only orthodox religion followers during the fasting season in which feeding habits might be reduced either in food diversity, especially animal source foods, or meal frequency which underestimate the finding when compared to other periods. Also, the above study was conducted in the dry season what we call “winter” in which the nutritional availability of most fruits and vegetables might be low compared to seasons especially “summer” a period in which this study was conducted.

This finding was also higher than the EDHS report of 2016, only 7% of children aged 6–23 months received a minimum acceptable diet [2]. The difference might be due to EDHS were conducted on a culturally different population, which may underrate child feeding practices while this study was conducted on an almost culturally homogenous population with similar feeding practices. The results of this study were higher than studies conducted in Ethiopia multilevel analysis report of EDHS 2016 (6.1%), Malawi (8.36%), Nigeria (7.3%), and Philippines (6.7%) of children aged 6–23 months received the recommended minimum acceptable diet [9, 10, 14–16]. The reason for a high percentage of feeding practice in this study area might be due to variation in study design, data collection period, and nutrition education with media and health extension workers might play a major role in increasing community awareness towards appropriate child feeding practice [17].

On the other hand, the finding of this study was lower than a study done in wolayita Sodo town (Southern Ethiopia) 21.1% of children consumed a minimum acceptable diet [18]. The variation might be because of different study settings and study periods; this study was conducted in the rural communities whereas the above study was conducted in urban communities, as communities from rural areas are less likely to feed a minimum acceptable diet than people residing in the urban area [2]. Also, the difference might be due to higher non-educated mothers were participated in this study, on the contrary, higher numbers of educated participants were included in the above study. The result was also lower than the study conducted in different countries; Ghana, Uganda, and Kenya in which 29.9, 23.9, and 48.5% of children received recommended minimum acceptable diet, respectively [19–21]. Lower findings in this study area might be due to differences in study design, sample size, study period, and difference in socio-demographic characteristics. Also, the finding was low compared to the 2020 global nutrition report (18.9%) [22]. The variation might be due to difference in sample size and socio-demographic characteristics.

Mother education was significantly associated with minimum acceptable diet practice. Based on this study, mothers who had formal education were 2.7 times more likely to provide minimum acceptable diets for their children compared to mothers who had no formal education. This finding was supported by a study done in Dembecha [10]. This might show that education enables mothers to know the benefits of the practice of child feeding and plays an important role in meeting minimum acceptable diet standards. However, this finding was not supported by the study done in North Shoa, Oromia region, and multilevel analysis report of EDHS 2016 [9, 23].

Children born in a health facility were 4.5 times more likely to receive a minimum acceptable diet than those born at home. This result was similar to a study done in Northwest Ethiopia [10]. This might be due to health professional counseling on appropriate child feeding after delivery on health facility increases mothers awareness on practice of minimum acceptable diet; Hence mother’s awareness on appropriate child feeding practice who got from health professionals have had a better child feeding practices than their counterparts [17].

Children whose mothers were exposed to media i.e. watched television, listen to the radio every day or once a week, has 2.6 times more likely to meet the minimum acceptable diet than those children of mothers who watched television and listen to the radio less than once a week or not at all. This finding was similar to other findings in North Shoa, Oromia region, and multi-level analysis report of EDHS 2016 [9, 23]. This might be because the currently Ethiopian ministry of health and its partners promote child feeding practices through radio, television, and family health cards. This might enhance the mother’s awareness of feeding a minimum acceptable diet to their children. Also, this might be because mothers who have been exposed to the media have had better opportunities to access information on appropriate child feeding practices. This could improve the mother’s capacity to challenge unfavorable information towards child feeding practices in the community and increase appropriate child feeding habits. On the other hand, this finding was not supported by the study conducted in Dembecha [10].

Children born from mothers with a high wealth index were 2.5 times more likely to receive the recommended minimum acceptable diet than children born from mothers with a low wealth index. This result was nearly similar to a study done in the Philippines [24], in which children born from mothers in the middle wealth index were more likely to meet the minimum acceptable diet compared to those children born from mothers in the poorest wealth index, The possible explanation of this significant association might be due to the limited food purchasing power to provide diversified diet to their children in peoples with lower wealth index, and also mothers in high wealth index were more likely provide nutritious food to their children compared to mothers from low wealth index households who were more focus on the quantity of food [11]. This finding was not supported by other studies conducted in Dembecha, North Shoa, and the multilevel analysis report of EDHS 2016 [9, 10, 23].

### Limitation

This study only measures the diversity and frequency of foods given for children aged 6–23 months, but it is important to include the quantity of foods given for the child. Even if children who were sick were excluded, there might be children who were not known to sick or not sick in the previous one week, but who lost their appetite during data collection time, which could underestimate our finding. This study didn’t consider seasonal variations during the data collection period, which might be affected feeding habits especially food diversity be affected.

## Conclusion

Minimum acceptable diet practice among children aged 6–23 months in the study area was low, almost one from eight children meet the recommended minimum criteria. So, child feeding practices in the study area were not achieved the national infant and young child feeding recommendation. Determinant Factors that significantly affect meeting of minimum acceptable diet practice were the educational status of the mother, place of delivery, exposure to media, and household wealth index. This implies that the problems are range from individual to household level, and even may go through at large in the community level.

## Data Availability

The datasets used during the current study are available from corresponding authors when reasonably desired.

## Abbreviations

AOR: Adjusted Odd Ratio
COR: Crude Odd Ratio
EDHS: Ethiopian Demographic Health Survey
IYCF: Infant and young children feeding
MAD: Minimum Acceptable Diet
SPSS: Statistical Package for Social Science
UNICF: United Nations International Children Fund
WHO: World Health Organization
